# Esophageal Pressure and Clinical Assessments in the Gastroesophageal Reflux Disease Patients with Laryngopharyngeal Reflux Disease

**DOI:** 10.3390/jcm10225262

**Published:** 2021-11-12

**Authors:** Yung-An Tsou, Sheng-Hwa Chen, Wen-Chieh Wu, Ming-Hsui Tsai, David Bassa, Liang-Chun Shih, Wen-Dien Chang

**Affiliations:** 1 Department of Otolaryngology-Head and Neck Surgery, China Medical University Hospital, Taichung 40402, Taiwan; d22052121@gmail.com (Y.-A.T.); minghsui5121@gmail.com (M.-H.T.); entdrshih7111@gmail.com (L.-C.S.); 2 Department of Audiology and Speech-Language Pathology, Asia University, Taichung 41354, Taiwan; shchen@asia.edu.tw; 3 School of Medicine, China Medical University, Taichung 40402, Taiwan; 4 Gastro Esophageal Reflux Disease (GERD) Center, Yuan Sheng Hospital, Changhua 51053, Taiwan; wenchiehwu0909@gmail.com; 5 Department of Biology, Lamar University, Beaumont, TX 77707, USA; davidbassa@hotmail.com; 6 Graduate Institute of Biomedical Sciences, China Medical University, Taichung 40402, Taiwan; 7 Department of Sport Performance, National Taiwan University of Sport, Taichung 404401, Taiwan

**Keywords:** laryngopharyngeal reflux disease, gastroesophageal reflux disease, esophageal pressure

## Abstract

Laryngopharyngeal reflux disease (LPRD) might be associated with reflux symptoms, and its severity is correlated with the Reflux Symptoms Index. Diagnosis is often challenging because of a lack of accurate diagnostic tools. Although an association between LPRD and gastroesophageal reflux disease (GERD) exists, the extent to which esophageal pressure changes in patients with LPRD with GERD has been unknown. Therefore, this study surveys the clinical assessments and extent of esophageal pressure changes in LRPD patients with various GERD severities, and compares esophageal sphincter pressures between ages, genders, and body mass index (BMI). This observational study assessed patients with LPRD and GERD. High-resolution esophageal manometry was used to gather data pertaining to the area pressure on the upper esophageal sphincter (UES) and lower esophageal sphincter (LES), and the correlation between such pressure and symptom severity was determined. We compared the esophageal pressure of different UES and LES levels in the following categories: gender, age, BMI, and GERD severity. We analyzed correlations between esophageal pressure and clinical assessments among 90 patients with throat globus with laryngitis with LPRD. LPRD was measured using laryngoscopy, and GERD was measured using esophagoscopy and 24 h PH monitoring. There were no significant differences in the clinical assessments among the four grades of GERD. The LPRD patients with serious GERD had a lower UES and LES pressure. The lowest pressure and longer duration of LES and UES were also observed among patients with LPRD of grade D GERD. No significant differences in UES and LES pressures among ages, genders, or BMIs were noted.

## 1. Introduction

Chronic cough, a globus sensation with dysphagia, and irritable throat discomfort can be bothersome symptoms for patients. These symptoms can present in patients with gastroesophageal reflux disease (GERD) as extraesophageal symptoms. They can also occur in patients with laryngopharyngeal reflux disease (LPRD) but not severe GERD [1]. LPRD occurs when stomach acid and digestive enzymes travel to the esophagus and then to the laryngopharynx to cause irritation and damage [2]. The condition can be diagnosed by using laryngoscopy, reflux finding scores (RFS), and the reflux symptom index (RSI) [3]. The relationship between GERD and LPRD is not well understood. In recent years, LPRD and GERD have been considered two distinct diseases although considerable overlap between the two diseases exists [4]. This overlap may lead to an underestimation of LPRD incidence.

GERD typically manifests as heartburn, regurgitation, and chest pain that result from lower esophageal sphincter (LES) dysfunction [5]. By contrast, patients with LPRD usually do not report these symptoms and are instead concerned about chronic cough, a globus sensation with laryngitis, sensation of a lump in the throat, and laryngitis due to upper esophageal sphincter dysfunction [6]. Incompetent antireflux barriers at the esophagogastric junction cause gastroesophageal reflux to become GERD. Antireflux is a functional mechanism of the LES. Relative to patients without GERD, patients with GERD have an LES pressure that is lower than the intragastric pressure, but also have a higher frequency of transient LES relaxation.

LPRD is considered esophageal sphincter dysfunction and often causes gastric-content reflux to ascend the upper esophageal sphincter (UES) [7]. As many as 60% of patients with GERD present frequent LPRD symptoms [8]. One study indicated that patients with LPRD had higher levels of anxiety and depression and lower quality of life relative to those with GERD without LPRD [9]. There was unknown esophagus pressure in patients with both LPRD and GERD, which is most difficult to treat. We scoped patients with LPRD by using of laryngoscopy graded by RFS (reflux fiberscopic scales) and GERD confirmed by use of esophagoscopy and 24 h PH monitoring, who were concerned about having a lump in their throat. Subsequently, the aims of our study were (1) to see the esophagus pressure by esophageal manometry in LPRD patients with various grades of GERD; and (2) to compare the sphincter pressures between patient-related factors (age, gender, and BMI).

## 2. Methods

### 2.1. Study Procedures

This was an observational study, and clinical diagnosis and standard assessments were conducted at the department of Otolaryngology-Head and Neck Surgery. The patients’ data were enrolled under Institutional Review Board, and performed per the Helsinki Declaration and the guidelines of the department. In total, the assessment data to determine that 109 patients with severe globus and with laryngitis; diagnosed using laryngoscopy to have LPRD revealed prominent arytenoid swelling, pseudosulcus, and laryngeal edema with RFS score > 7 and RSI > 13, and these instances of LPRD were also confirmed using 24-h impedance-pH monitoring. LPRD is defined as a PH < 4 detected from 3–5 cm below the hypopharyngeal area for more than 4 times per day [10,11]. Subsequently, the 109 patients with LPRD who underwent esophagoscopy with 24-h impedance-pH monitoring above the esophageal gastric junction and we define the PH < 4 and > 4% test time as GERD. In addition, those who the esophagogastroscopy also revealed according to Los Angeles (LA) a classification of at least ‘A’ were enrolled in our study. There were a total 90 patients with LPRD and GERD that underwent complete esophageal manometry, and the data gathered were used to determine the correlation of esophageal pressure with LPRD and GERD. Clinical assessments (i.e., the RSI, Eating Assessment Tool-10 (EAT-10), and RFS [12]) were also conducted to record by an experienced otolaryngologist and speech-language pathologist. The so-called mucosal break was used to describe lesions at the junction of the esophagus and gastric inlet. The erythema, or sloughing mucosa, is separated from the normal mucosa at the EG junction. GERD severity was classified into four grades (grades A–D) according to the mucosa break’s severity.

### 2.2. Esophageal Pressure Assessment

**Figure 1:**
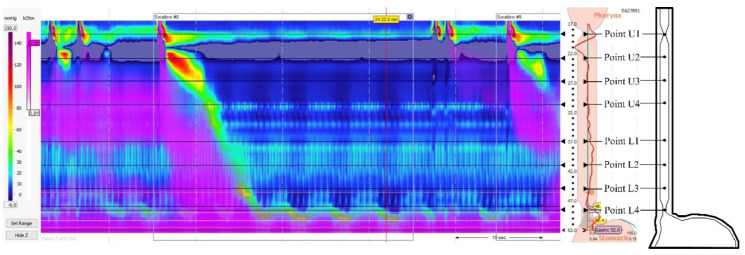
Illustration of esophageal pressure assessment points and normal pressure pattern finding in high-resolution esophageal manometry.

High-resolution esophageal manometry (ManoView, Sierra Scientific, Los Angeles, CA, USA) was used to assess esophageal motility patterns by measuring swallowing in the esophagus. Data were gathered at four points in the UES area and four points (point U1–U4) in the LES (points L1–L4; Figure 1). The catheter was placed to span the esophagus’ length, and the distal sensor was positioned 3 cm below the diaphragm. The patients underwent a 30-sec quiescent period for baseline data to be gathered. Each participant was given water and an edible object to swallow in a reclined position [13]. Swallowing begins with the relaxation of the UES and contraction of the LES along the esophagus’ length, and the esophageal pressure is then restored. Regular pressure patterns and reflux during swallowing are represented in Figure 1. The patients consumed 5 mL of room-temperature water 10 times and then ate the edible object five times at the same amount and temperature. The data were collected during swallowing on the average esophageal pressure at eight points dispersed along a standard pressure pattern.

### 2.3. Clinical Assessments

#### 2.3.1. Reflux Finding Score

The RFS is an eight-item index that includes pseudosulcus vocalis, ventricular obliteration, erythema/hyperemia, vocal fold edema, diffuse laryngeal edema, posterior commissure hypertrophy, granuloma/granulation, and thick endolaryngeal mucus [14]. It is designed to assess clinical severity based on laryngoscopic findings. Scores range from 0 (normal) to 26 (most severe), with a score of 7 or above being generally considered to indicate LPRD. All RFS is scored by two senior laryngologists arriving at consensus during scoring.

#### 2.3.2. Eating Assessment Tool-10

EAT-10 is a questionnaire for assessing symptom severity and quality of life in patients with oral–pharyngeal dysphagia [15]. The 10 items of EAT-10 were arranged on a five-point scale. The score indicates the severity level (0: no problem; 1: a slight problem; 2: a mild problem; 3: a moderate problem; and 4: severe problem).

#### 2.3.3. Reflux Symptom Index

The RSI is a nine-item self-administered outcome questionnaire designed to document LPRD symptoms and severity [16]. Patients were asked to rate how nine problems have affected them over the past month on a scale of 0 (no problem) to 5 (severe problem) with a maximum total score of 45. A total score of more than 13 indicates a diagnosis of LPRD.

### 2.4. Statistical Analysis

SPSS software (version 19, SPSS, Chicago, IL, USA) was used for statistical analysis. Descriptive statistics were presented, and nonparametric statistics were used to compare differences between and within groups. One-way analysis of variance (ANOVA) was used to determine the statistical differences among the groups. Tukey’s test and a post hoc test were used to determine the difference between the two groups. The basic characteristics of enrolled patients, such as their age and BMI, were collected for a subgroup analysis on LES and UES pressure. The age groups were <39 years, 40–49 years, 50–59 years, and >60 years. Consistent with World Health Organization guidelines, the lower cutoff values were recommended as 28 kg/m^2^ for the East Asian population, and BMI  ≥  28 kg/m^2^ indicated obesity [17]. A *p*-value < 0.05 was considered statistically significant.

## 3. Results

All 90 patients with both LPRD and GERD were enrolled, and Table 1 lists the patients’ baseline characteristics. Among them, 83 patients had gastritis (92.22%) and 81 patients had esophagitis (90%). The mean RFS > 7 and mean RSI > 13 of all patients were determined. All patients completed the esophageal pressure and clinical assessments. The assessment results were grouped by LA classification: 27 patients (30%) into grade A, 33 patients (36.67%) into grade B, 17 patients (18.89%) into grade C, and 13 patients (14.44%) into grade D. With regard to LES pressure, the changes in duration and LES pressure were significant among the four groups (*p* < 0.05).

### 3.1. Changes on Esophageal Pressure in LRPD Patients with Various GERD Severity

The results of ANOVA are summarized and showed in Table 2. The differences in RFS, EAT-10, and RSI were not found among the four grades of GERD (all *p* > 0.05). Comparing the UES and LES duration, significant differences were noted among the different grade GERD (*p* < 0.05). We also found that the LES and UES durations, point U 2-4 of UES pressure and point L1-2 of LES pressure, considerably differed among the four grades of GERD (all *p* < 0.05). The post hoc tests revealed that grade D of GERD had the lowest LES and UES durations among four GERD grades (Table 3). Comparing to grade A of GERD, the patients with grade D of GERD also significantly had the lowest esophageal pressure at point U 1–4 of UES, and point L 1–4 of LES.

A trend of higher UES and LES pressures in slight grade of GERD were found in Table 3. In addition, the patients with simultaneous LPRD and GERD presented a trend in which UES pressure was higher than LES pressure. The lowest pressure was also observed in patients with LPRD with grade D GERD. Therefore, esophagus pressure change really correlated in patients with both LPRD and GERD, and the lowest UES and LES pressures were in GERD D patients. The pressure is noted higher both nearest to UES (U1) and nearest to LES (L4) in patients diagnosed to have both LPRD and GERD in our study.

### 3.2. Results of Comparing Sphincter Pressures across Age, Gender, and BMI

With regard to the esophageal pressure for each of the four LES or UES points, LES and UES pressures did not significantly differ between ages, genders, and BMIs (*p* > 0.05, Table 4). We noted that a trend of lower UES and LES pressures was accompanied by age. However, trends of esophageal pressure differences in different genders and BMIs were not found.

## 4. Discussion

In this study, the patients with LPRD with GERD, esophageal pressure (specifically UES and LES pressure) was assessed and symptom severity was indicated by the RFS, EAT-10, and RSI. UES and LES pressure did not differ between genders, ages, and BMIs. The esophagus pressure change really correlated with in LPRD and GERD patients, and the lowest pressure was found in severe GERD patients. However, the UES pressure (U1–U4), and LES pressure (L1–L4) was substantially lower in severe GERD (*p* < 0.05). We also analyzed the relationship of RFS, EAT-10, and RSI between GERD severity (A, B, C, D), but no significant correlation is found. Thus, there were really lower UES and LES in patients with LPRD and GERD, and the lowest pressure is found in GERD D patients with LPRD.

LPRD and GERD are similar but must never be treated as identical. GERD typically manifests as heartburn, regurgitation, and chest pain [1]. Patients with LPRD usually do not report these symptoms and are concerned instead about chronic cough, a lump in the throat, and laryngitis [18]. LPRD and GERD also differ with respect to the timing of reflux. In GERD, reflux and acidity typically occur at night, whereas in LPRD reflux occurs during the day. LPRD symptoms occur when patients are upright, whereas GERD reflux occurs when patients are in the supine position. Dysfunction in the UES and LES also plays an essential role in the disease. Patients with GERD experience dysfunction in the LES, which allows stomach acid to move up the esophagus and cause heartburn [19]. In patients with LPRD dysfunction in the UES, stomach acids and enzymes travel up the throat [20]. Although a correlation has been identified between the presence of LPRD and the severity of GERD, such a correlation may lead to underestimation of the incidence of LPRD [21].

Laryngopharyngeal reflux might also have esophageal symptoms related to GERD. However, the relationship between these two conditions has not been studied. GERD could be diagnosed using multiple tools, such as esophagoscopy, esophagogastroscopy, and 24 h of pH monitoring [22]. By contrast, fewer objective diagnostic tools exist for accurately diagnosing LPRD. The detection of pepsin in the larynx or pharynx has been reported to indicate an acid reflux event, and this scenario is why LPRD is often referred to as ‘silent’ reflux. The mucosa in the upper esophagus, compared with the lower esophagus, which is less resistant, is more sensitive to acid and pepsin exposure. Therefore, patients with LPRD may present with chronic cough, asthma, or laryngitis but not typical GERD-related symptoms; this discrepancy makes diagnosis difficult [23]. Additionally, signs of gastroesophageal reflux can be found in the laryngopharynx of up to 86% of healthy individuals, and this circumstance makes the diagnosis of LPRD even more difficult [24].

Studies have reported that a globus sensation with laryngitis is correlated with GERD with lower LES and UES pressures, as measured using esophageal manometry. Normal esophageal deglutition is maintained by proper pressure in the UES and LES with acceptable relaxation and by proper peristalsis of the esophageal muscles. During swallowing, the intrabolus pressures in the UES and LES were 32–140 and 43–92 mmHg respectively in our study. The lower LES is correlated with GERD. In addition, LPRD and pharyngeal dysphagia is often associated with a decreased UES pressure [25]. Decreased LES pressure in patients with GERD is also suggested, as is a trend of LES decreasing with an increase in GERD grade. However, few reports have indicated that the changed UES is associated with LPRD. Nonetheless, we found decreased UES pressure in patients with GERD with throat globus sensation with laryngitis. We also observed that UES pressure was lower in LPRD patients with a severe GERD grade. The assessment tools—i.e., RFS, EAT-10, RSI—are found no correlates to the severity of GERD. In our findings, the UES and LES pressure are both higher in more severe GERD grades. Our findings also revealed a trend of lowest pressure found near UES (U1) and LES (L4) in patients with simultaneous LPRD and GERD, the trend is more remarkable in patients with GERD D group.

A lack of standard diagnostic testing for LPRD exists. LPRD is most often misdiagnosed as GERD by physicians unfamiliar with the differences between LPRD and GERD [24]. RFS score can be used to quantify laryngeal inflammation with 95% sensitivity. However, the specificity is low because this inflammation may be a result of infections, allergies, or other causes unrelated to LPRD [24]. The GERDQ, a questionnaire designed to quantify GERD, has a specificity of 71.4% and a sensitivity of 64.6% [26]. In our study, we observed a substantial decrease in UES pressure in cases of severe GERD. Decreased UES and pharyngeal pressures are also infrequently reported in patients with globus with laryngitis. Therefore, severe globus with laryngitis might be present in patients with severe GERD diagnoses classified as LA GERD C or D. Therefore, we intend to use esophageal manometry as an additional objective measurement for grading the severity of LPRD. We found that the UES is lowest in GERD D. The LES esophagus pressure is found lowest in GERD D. The UES and LES pressure with higher duration is lowest in GERD D patients. However, not all of the pressure is significantly different between each of the GERD groups. Our study is like other few pioneer studies to diagnose the LPRD and GERD by measuring the UES and LES PH monitoring [27,28], and check all of their esophageal manometry to survey the pressure changes between LPRD and GERD.

Other studies that have measured resting LES values in GERD patients with LPRD, or healthy individuals, have reported no substantial differences in pressure with a baseline of 15 mmHg [29]. There was a study reported no considerable difference between LES resting pressure for patients with LPRD; however, a considerable difference is present regarding UES dysfunction [30]. Our study found that lower LES pressure is correlated with RSI but not with RFS and EAT-10. This finding supports the fact that RSI was designed for patients with GERD [31]. However, RFS is scaled according to pharynx and larynx laryngoscopic results for patients with LPRD; LES’s lesser correlation is presumed. RFS is subjective and might have an interrater or intrarater difference in the small sample size of the study. EAT-10 is designed for LPRD and could be helpful to predict the severity of LPRD because LPRD is considered a pressure disorder of UES; this finding indicates a smaller correlation between EAT-10 and LES pressure. An exciting finding in the literature is that UES pressure disorder is not correlated with RSI, RFS, or EAT-10, even though UES pressure disorder is considered correlated with RFS or EAT-10 [32,33,34]. Herbella et al. indicated that the otorhinolaryngologic symptoms and laryngoscopy were unreliable assessments for GERD diagnosis [27]. These results were similar to our findings that the patients with LPRD and GERD did not have significant differences in RFS, EAT-10, and RSI between the different grades of GERD. We also did not find correlations between these clinical assessments and GERD severity (GERD A-D). In addition, whether esophagus pressure changes associated with age, gender, BMI was widely debated, and there were still few studies focused on the age, gender, and BMI effects on pressure changes in patients with both GERD and LPR. However, we did not find differences of pressure changes across age, gender, and BMI.

**Figure 2:**
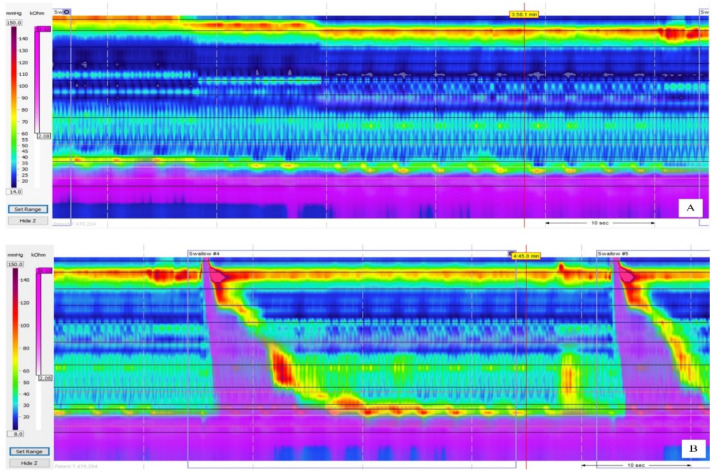
Illustration of esophageal pressure pattern during stationary (**A**) and swallowing (**B**) stages in grade D GERD.

One study demonstrated that a higher cricopharyngeal bar rate with elevated pharyngoesophageal segment pressure is associated with a higher stricture rate by hypertrophy of the cricopharyngeal muscle [32]. Such elevated pressure can be corrected through the use of proton pump inhibitors (PPIs), balloon dilatation, or botox injection or by excision of the cricopharyngeal bar. Some studies have indicated that people with LPRD with GERD might have lower UES pressure in the early stages and that those pressures increase over time [32,33,34]. In our findings, decreased LES pressure is correlated with the severity of GERD. Lower LES pressure is more common in grade D GERD (Table 4 and Figure 2). Severe exposure to acid or severely damaged EG junction mucosa might be caused by lower LES sphincter pressure. Several studies have found that lower LES pressure in the human esophagus prevents further acid mucosa injury to the esophagus [35,36,37,38]. We also found lower UES and LES pressure in patients with LPRD with severe GERD.

### Study Limitation

The diagnosis for LPRD proved by PH < 4 for over four times might overestimate the LPRD diagnostic rate. Although, RSI, RFS, and EAT-10 were used as additional diagnostic tools. These methods, however, have low specificity. GERD defined by PH < 4 for over 4% test time might also overestimate the GERD diagnostic rate but be supported by esophagogastroscopy findings according to LA classification. We still found that decreased LES in patients with GERD leads to decreased UES pressure, but the clinical meanings are difficult to conclude. In addition, this study is limited by the overlap between LPRD and GERD and we barely enrolled healthy individuals to form a control group but instead included patients with mild GERD as the control group.

## 5. Conclusions

Esophageal pressure changes with the severity of GERD in LPRD patients. No significant differences in the clinical assessments were noted among the different grades of GERD. Severe GERD presents a lower LES pressure on high-resolution manometry. Concurrently, lower UES is also associated with more severe GERD grading conditions. Patients with LPRD diagnosed in our study had a trend of simultaneous reduction in pressure in UES, and the more pressure reduction is also associated with more severe GERD condition. The grade D GERD was associated with the lowest LES pressure. We also found the lowest UES pressure among patients with grade D GERD who were also diagnosed with LPRD. Age, gender, and BMI were not relevant factors with respect to pressure changes in esophageal pressure.

## Figures and Tables

**Table 1:** Patient’s baseline characteristics.

Items	Patients (*n* = 90)
Male/Female	41/49
Age (years)	51.57 ± 13.63
BMI (kg/m^2^)	27.75 ± 7.51
No. of BMI ≥ 28, *n* (%)	22 (24.4%)
No. of patients with gastritis, *n* (%)	82 (91.1%)
No. of patients with esophagitis, *n* (%)	81 (90.0%)
RFS	11.05 ± 3.64
EAT-10	6.08 ± 7.35
RSI	20.76 ± 7.35

BMI: body mass index; RFS: reflux finding scores; EAT-10: Eating assessment tool-10; RSI: reflux symptom index.

**Table 2:** Comparison pressure of different grades in GERD.

	Grade A (*n* = 27)	Grade B (*n* = 33)	Grade C (*n* = 17)	Grade D (*n* = 13)	F Value	*p*
RFS	10.25 ± 5.64	13.41 ± 7.02	11.01 ± 8.74	11.09 ± 9.65	1.01	0.39
EAT-10	5.73 ± 6.34	6.64 ± 8.36	7.38 ± 8.34	7.52 ± 9.12	0.22	0.87
RSI	21.26 ± 6.83	20.76 ± 6.83	21.83 ± 8.95	23.92 ± 9.87	0.54	0.65
UES duration (s)	2.42 ± 0.55	2.44 ± 0.71	3.27 ± 0.72	3.67 ± 0.34	19.21	0.001 *
UES Pressure (mmHg)						
Point U1	140.12 ± 39.37	127.05 ± 36.53	114.28 ± 40.57	113.06 ± 32.50	2.02	0.11
Point U2	126.34 ± 29.68	95.57 ± 37.51	93.05 ± 35.38	88.08 ± 23.53	4.54	0.01 *
Point U3	104.22 ± 37.40	64.55 ± 21.67	64.06 ± 20.76	40.05 ± 24.72	19.32	0.001 *
Point U4	102.67 ± 29.96	39.08 ± 26.38	37.02 ± 19.57	32.01 ± 18.89	33.31	0.001 *
LES duration (s)	1.51 ± 0.65	2.26 ± 0.62	4.73 ± 1.50	6.75 ± 1.87	87.67	0.001 *
LES Pressure (mmHg)						
Point L1	77.05 ± 21.26	62.05 ± 18.32	60.03 ± 27.16	43.34 ± 19.90	22.74	0.001 *
Point L2	79.03 ± 21.63	70.08 ± 19.05	66.08 ± 27.17	52.56 ± 25.95	7.21	0.001 *
Point L3	87.06 ± 32.62	76.51 ± 37.78	62.24 ± 30.98	60.09 ± 28.12	2.61	0.06
Point L4	92.01 ± 35.19	83.04 ± 32.09	82.11 ± 34.01	69.12 ± 36.09	1.35	0.26

* *p* < 0.05; UES, upper esophageal sphincter; LES, lower esophageal sphincter; RFS, reflux finding scores; RSI, reflux symptom index; EAT-10, eating assessment tool-10.

**Table 3:** Summary of *p*-values of post hoc test in assessment values between the two grade of GERD.

	Grade A vs. Grade D	Grade B vs. Grade A	Grade C vs. Grade B	Grade D vs. Grade C	Comparison
RFS	0.72	0.06	0.30	0.98	B > D > C > A
EAT-10	0.47	0.64	0.76	0.96	C > D > B > A
RSI	0.32	0.77	0.63	0.54	B < A < C < D
UES duration (s)	0.001 *	0.90	0.001 *	0.07	A < B < C < D
UES Pressure (mmHg)					
Point U1	0.03 *	0.18	0.26	0.92	A > B > C > D
Point U2	0.001 *	0.001 *	0.81	0.66	A > B > C > D
Point U3	0.001 *	0.001 *	0.93	0.007 *	A > B > C > D
Point U4	0.001 *	0.001 *	0.77	0.48	A > B > C > D
LES duration (s)	0.001 *	0.001 *	0.001 *	0.002 *	A < B < C < D
LES Pressure (mmHg)					
Point L1	0.001 *	0.004 *	0.78	0.04 *	A > B > C >D
Point L2	0.001 *	0.09	0.50	0.17	A > B > C >D
Point L3	0.01 *	0.25	0.18	0.84	A > B > C > D
Point L4	0.02 *	0.30	0.92	0.32	A > B > C > D

* *p* < 0.05; UES, upper esophageal sphincter; LES, lower esophageal sphincter; RFS, reflux finding scores; RSI, reflux symptom index; EAT-10, eating assessment tool-10.

**Table 4:** Comparison of pressure of different LES levels across gender, age, and BMI.

Factors (No. of Patient)	LES Duration (s)	LES Pressure (mmHg)				UES Duration (s)	UES Pressure (mmHg)			
		Point L1	Point L2	Point L3	Point L4		Point U1	Point U2	Point U3	Point U4
Gender (*n* = 90)										
Female (*n* = 49)	2.51 ± 2.33	61.42 ± 50.45	63.87 ± 66.32	80.85 ± 80.12	87.67 ± 90.55	2.63 ± 2.44	127.42 ± 100.23	97.40 ± 89.32	55.75 ± 58.32	36.35 ± 38.34
Male (*n* = 41)	2.56 ± 2.76	69.46 ± 60.11	68.85 ± 63.21	74.15 ± 80.77	76.86 ± 69.28	2.54 ± 2.45	112.35 ± 89.32	97.69 ± 91.33	65.19 ± 64.78	44.31 ± 56.32
	*p* = 0.75	*p* = 0.13	*p* = 0.34	*p* = 0.07	*p* = 0.64	*p* = 0.85	*p* = 0.14	*p* = 0.63	*p* = 0.45	*p* = 0.17
Age (*n* = 90)										
<39 (*n* = 18)	2.73 ± 2.33	70.53 ± 79.87	74.03 ± 69.56	86.82 ± 71.21	96.37 ± 90.12	2.63 ± 2.38	128.52 ± 101.78	119.42 ± 97.89	70.09 ± 72.32	53.23 ± 60.21
40~49 (*n* = 23)	1.84 ± 3.01	64.12 ± 57.91	66.85 ± 66.01	84.61 ± 90.12	85.83 ± 78.34	2.87 ± 2.56	126.60 ± 102.52	101.86 ± 98.87	56.98 ± 47.21	35.56 ± 36.77
50~59 (*n* = 28)	2.63 ± 2.89	62.87 ± 61.23	66.12 ± 62.89	80.16 ± 79.28	84.22 ± 80.23	2.33 ± 2.32	123.10 ± 98.35	99.74 ± 98.22	54.66 ± 47.55	38.19 ± 36.70
>60 (*n* = 21)	2.67 ± 3.21	58.29 ± 55.22	59.07 ± 62.45	74.81 ± 76.56	78.33 ± 69.43	2.97 ± 3.32	109.66 ± 70.21	88.67 ± 90.32	46.09 ± 50.44	28.51 ± 47.56
	*p* = 0.82	*p* = 0.72	*p* = 0.67	*p* = 0.49	*p* = 0.85	*p* = 0.94	*p* = 0.50	*p* = 0.35	*p* = 0.56	*p* = 0.41
BMI (*n* = 90)										
<28 (*n* = 55)	2.35 ± 2.66	60.72 ± 58.46	63.17 ± 62.38	74.82 ± 69.76	80.21 ± 79.33	2.67 ± 2.35	115.82 ± 89.45	97.59 ± 85.39	66.25 ± 50.19	37.42 ± 35.77
≥28 (*n* = 35)	1.86 ± 3.01	68.75 ± 59.32	71.81 ± 69.88	75.62 ± 72.43	94.25 ± 87.56	2.70 ± 2.76	105.60 ± 70.32	96.40 ± 88.24	58.21 ± 55.84	44.23 ± 46.93
	*p* = 0.52	*p* = 0.21	*p* = 0.90	*p* = 0.88	*p* = 0.62	*p* = 0.12	*p* = 0.42	*p* = 0.64	*p* = 0.89	*p* = 0.88

UES, upper esophageal sphincter; LES, lower esophageal sphincter; BMI; body mass index.

## Abbreviations

LPRD	laryngopharyngeal reflux disease
GERD	gastroesophageal reflux disease
UES	upper esophageal sphincter
LES	lower esophageal sphincter
RFS	reflux finding scores
RSI	reflux symptom index
BMI	body mass index
EAT-10	eating assessment tool-10
LA	Los Angeles
PPIs	proton pump inhibitors
H2	histamine-2
